# 1-Chloro­acetyl-*r*-2,*c*-6-bis­(4-methoxy­phen­yl)-*t*-3-methyl­piperidin-4-one

**DOI:** 10.1107/S1600536809043281

**Published:** 2009-10-28

**Authors:** K. Ravichandran, P. Ramesh, P. Sakthivel, S. Ponnuswamy, M. N. Ponnuswamy

**Affiliations:** a Centre of Advanced Study in Crystallography and Biophysics, University of Madras, Guindy Campus, Chennai 600 025, India; b Department of Chemistry, Government Arts College (Autonomous), Coimbatore 641 018, India

## Abstract

There are two crystallographically independent mol­ecules in the asymmetric unit of the title compound, C_22_H_24_ClNO_4_. The piperidine ring in both mol­ecules adopts a distorted boat conformation. The crystal packing is stabilized by C—H⋯O and C—H⋯Cl inter­actions.

## Related literature

For ring puckering parameters, see: Cremer & Pople (1975 ▶); Nardelli (1983 ▶). For the pharmacological properties of piperidin-4-ones, see: El-Subbagh, Abu-Zaid, Mahran, Badria & Al-obaid (2000 ▶); Ganellin & Spickett (1965 ▶); Hagenbach & Gysin (1952 ▶); Jerom & Spencer (1988 ▶); Katritzky & Fan (1990 ▶); Mobio *et al.* (1989 ▶); Perumal *et al.* (2001 ▶).
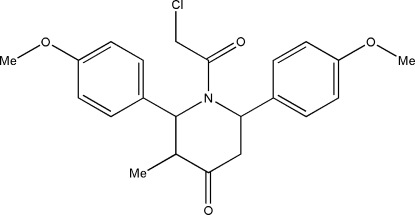


## Experimental

### 

#### Crystal data

C_22_H_24_ClNO_4_
*M* *_r_* = 401.87
Triclinic, 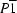

*a* = 8.9147 (8) Å
*b* = 11.9648 (10) Å
*c* = 19.1365 (16) Å
α = 99.729 (5)°
β = 93.665 (5)°
γ = 90.112 (6)°
*V* = 2007.5 (3) Å^3^
*Z* = 4
Mo *K*α radiation
μ = 0.22 mm^−1^
*T* = 293 K
0.15 × 0.15 × 0.14 mm

#### Data collection

Bruker Kappa APEXII area-detector diffractometer
Absorption correction: multi-scan (*SADABS*; Sheldrick, 2001 ▶) *T*_min_ = 0.968, *T*_max_ = 0.970
37036 measured reflections
9971 independent reflections
5739 reflections with *I* > 2σ(*I*)
*R*_int_ = 0.043

#### Refinement

*R*[*F* ^2^ > 2σ(*F* ^2^)] = 0.054
*wR*(*F* ^2^) = 0.164
*S* = 1.05
9971 reflections
512 parameters
H-atom parameters constrained
Δρ_max_ = 0.49 e Å^−3^
Δρ_min_ = −0.37 e Å^−3^

### 

Data collection: *APEX2* (Bruker, 2004 ▶); cell refinement: *SAINT* (Bruker, 2004 ▶); data reduction: *SAINT*; program(s) used to solve structure: *SHELXS97* (Sheldrick, 2008 ▶); program(s) used to refine structure: *SHELXL97* (Sheldrick, 2008 ▶); molecular graphics: *ORTEP-3* (Farrugia, 1997 ▶); software used to prepare material for publication: *SHELXL97* and *PLATON* (Spek, 2009 ▶).

## Supplementary Material

Crystal structure: contains datablocks global, I. DOI: 10.1107/S1600536809043281/bt5090sup1.cif

Structure factors: contains datablocks I. DOI: 10.1107/S1600536809043281/bt5090Isup2.hkl

Additional supplementary materials:  crystallographic information; 3D view; checkCIF report

## Figures and Tables

**Table 1:** Hydrogen-bond geometry (Å, °)

*D*—H⋯*A*	*D*—H	H⋯*A*	*D*⋯*A*	*D*—H⋯*A*
C6*A*—H6*A*⋯O1*B*^i^	0.98	2.53	3.350 (3)	142
C6*B*—H6*B*⋯O1*A*	0.98	2.54	3.363 (3)	142
C8*A*—H8*B*⋯Cl1*B*^i^	0.97	2.81	3.684 (3)	150

Symmetry code: (i) 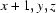.

**Fig. 1.**
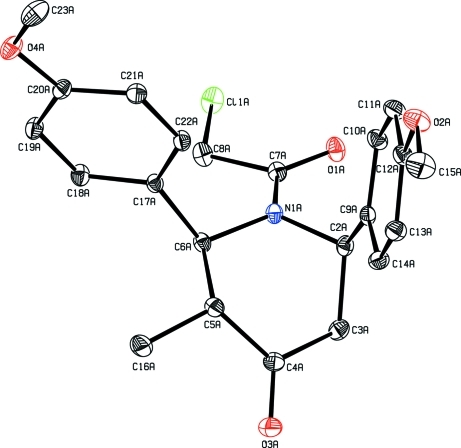
Perspective view of one of the two molecules in the asymmetric unit of the title compound showing displacement ellipsoids at 30% probability level. H atoms have been omitted for clarity.

**Fig. 2.**
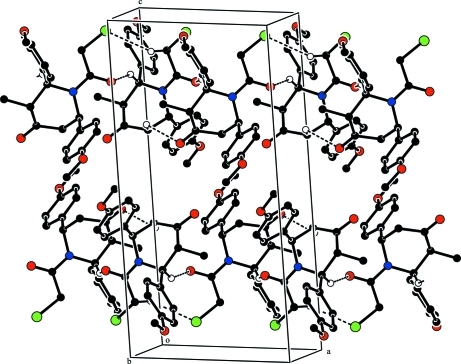
The crystal packing of the molecules viewed down b–axis. H atoms not involved in hydrogen bonding have been omitted for clarity.

## supplementary crystallographic information

### Comment

In the family of heterocyclic compounds, nitrogen containing heterocycles especially piperidin-4-ones presumably gaining considerable importance owing to their varied biological properties such as antiviral, antitumour (El-Subbagh  *et al.*, 2000), analgesic (Jerom *et al.*, 1988), local anaesthetic (Perumal *et al.*,  2001; Hagenbach & Gysin, 1952), antimicrobial, bactericidal, fungicidal, herbicidal, insecticidal, antihistaminic, anti-inflammatory, anticancer, *CNS* stimulant and depressant activities (Mobio *et al.*,  1989; Katritzky & Fan, 1990; Ganellin & Spickett, 1965). In view of these importance and to ascertain the molecular conformation, a crystallographic study of the title compound has been carried out.

The *ORTEP* diagram of the title compound is shown in Fig.1. There are two crystallographically independent molecules in the asymmetric unit. The piperidine ring in both the molecules adopts a distorted boat conformation with the puckering parameters (Cremer & Pople, 1975) and the asymmetry parameters (Nardelli, 1983) are: for molecule A: q_2_ = 0.654 (3) Å, q_3_ = -0.071 (2) Å, φ_2_ = 248.9 (2)° and Δ_s_(C2A & C5A)= 9.6 (2)°; for molecule B: q_2_ = 0.655 (3) Å, q_3_ = 0.065 (3) Å, φ_2_ = 69.3 (2)° and Δ_s_(C2B & C5B)= 10.0 (2)°. The sum of the bond angles around N atoms [N1A(358.2°) & N1B(357.9°)] of the piperidine ring in both the molecules are accordance with *sp*^2^ hybridization. One of the methoxy phenyl rings in both the molecules are approaximately orthogonal to the piperidine ring, which is evident from the interplanar angles of 87.6 (1)° & 88.3 (1)°, whereas the other rings are twisted by 76.3 (1) ° & 74.4 (1) °, respectively. The chloroacetyl group in each molecule is in extended conformation as can be seen from the torsion angles N1A—C7A—C8A—Cl1A (-179.7 (2)°) and N1B—C7B—C8B—Cl1B (-177.7 (1) °).

The crystal packing is stabilized by C—H···O and C—H···Cl interactions, which link the molecules into a chain extending along a–axis. Atoms C6A and C6B of the molecules at (*x*, *y*, *z*) donate one proton each to atoms O1B and O1A of the molecules at (*x* + 1, *y*, *z*) and (*x*, *y*, *z*), respectively, forming a C5 zig–zag chain running along a–axis.

### Experimental

To a solution of r-2,c-6-bis(4-methoxyphenyl)-t-3-methylpiperidin-4-one (1.625 g) in anhydrous benzene (60 ml) was added triethylamine (2.78 ml) and Chloroacetylchloride (1.59 ml). The reaction mixture was allowed to stirr at room temperature for 2hrs. The resulting solution was washed with sodium bicarbonate solution (10%), water and the organic layer was dried over anhydrous sodium sulfate, evaporated and crystallized from benzene: pet- ether (60–80°C) in the ratio of 95:05.

### Refinement

H atoms were positioned geometrically (C—H=0.93–0.98 Å) and allowed to ride on their parent atoms, with 1.5*U*_eq_(C) for methyl H and 1.2 *U*_eq_(C) for other H atoms.

### Crystal data

**Table:**

C_22_H_24_ClNO_4_	*Z* = 4
*M**_r_* = 401.87	*F*(000) = 848
Triclinic, *P*1	*D*_x_ = 1.330 Mg m^−^^3^
Hall symbol: -P 1	Mo *K*α radiation, λ = 0.71073 Å
*a* = 8.9147 (8) Å	Cell parameters from 4567 reflections
*b* = 11.9648 (10) Å	θ = 1.1–28.4°
*c* = 19.1365 (16) Å	µ = 0.22 mm^−^^1^
α = 99.729 (5)°	*T* = 293 K
β = 93.665 (5)°	Block, colorless
γ = 90.112 (6)°	0.15 × 0.15 × 0.14 mm
*V* = 2007.5 (3) Å^3^	

### Data collection

**Table:**

Bruker Kappa APEXII area-detector diffractometer	9971 independent reflections
Radiation source: fine-focus sealed tube	5739 reflections with *I* > 2σ(*I*)
graphite	*R*_int_ = 0.043
ω and φ scans	θ_max_ = 28.4°, θ_min_ = 1.1°
Absorption correction: multi-scan (*SADABS*; Sheldrick, 2001)	*h* = −11→11
*T*_min_ = 0.968, *T*_max_ = 0.970	*k* = −15→15
37036 measured reflections	*l* = −25→24

### Refinement

**Table:**

Refinement on *F*^2^	Secondary atom site location: difference Fourier map
Least-squares matrix: full	Hydrogen site location: inferred from neighbouring sites
*R*[*F*^2^ > 2σ(*F*^2^)] = 0.054	H-atom parameters constrained
*wR*(*F*^2^) = 0.164	*w* = 1/[σ^2^(*F*_o_^2^) + (0.0715*P*)^2^ + 0.4246*P*] where *P* = (*F*_o_^2^ + 2*F*_c_^2^)/3
*S* = 1.05	(Δ/σ)_max_ = 0.001
9971 reflections	Δρ_max_ = 0.49 e Å^−^^3^
512 parameters	Δρ_min_ = −0.37 e Å^−^^3^
0 restraints	Extinction correction: *SHELXL97* (Sheldrick, 2008), Fc^*^=kFc[1+0.001xFc^2^λ^3^/sin(2θ)]^-1/4^
Primary atom site location: structure-invariant direct methods	Extinction coefficient: 0.0045 (11)

### Special details

**Table:**

Geometry. All e.s.d.'s (except the e.s.d. in the dihedral angle between two l.s. planes) are estimated using the full covariance matrix. The cell e.s.d.'s are taken into account individually in the estimation of e.s.d.'s in distances, angles and torsion angles; correlations between e.s.d.'s in cell parameters are only used when they are defined by crystal symmetry. An approximate (isotropic) treatment of cell e.s.d.'s is used for estimating e.s.d.'s involving l.s. planes.
Refinement. Refinement of *F*^2^ against ALL reflections. The weighted *R*-factor *wR* and goodness of fit *S* are based on *F*^2^, conventional *R*-factors *R* are based on *F*, with *F* set to zero for negative *F*^2^. The threshold expression of *F*^2^ > σ(*F*^2^) is used only for calculating *R*-factors(gt) *etc*. and is not relevant to the choice of reflections for refinement. *R*-factors based on *F*^2^ are statistically about twice as large as those based on *F*, and *R*- factors based on ALL data will be even larger.

### Fractional atomic coordinates and isotropic or equivalent isotropic displacement parameters (Å^2^)

**Table:**

	*x*	*y*	*z*	*U*_iso_*/*U*_eq_	
Cl1A	0.69594 (8)	0.10702 (6)	0.06473 (4)	0.0702 (2)	
O1A	0.65647 (19)	0.09415 (15)	0.21138 (9)	0.0626 (5)	
O2A	0.7515 (2)	−0.36334 (16)	0.42043 (11)	0.0770 (6)	
O3A	1.2325 (2)	0.12592 (16)	0.38722 (10)	0.0706 (5)	
O4A	1.1117 (2)	−0.44641 (14)	0.05999 (10)	0.0697 (5)	
N1A	0.8822 (2)	0.02377 (14)	0.24329 (9)	0.0446 (4)	
C2A	0.8500 (3)	0.04894 (19)	0.31906 (12)	0.0494 (5)	
H2A	0.7556	0.0908	0.3214	0.059*	
C3A	0.9715 (3)	0.1301 (2)	0.35698 (14)	0.0578 (6)	
H3A	0.9639	0.2009	0.3388	0.069*	
H3B	0.9541	0.1463	0.4072	0.069*	
C4A	1.1273 (3)	0.08538 (18)	0.34847 (13)	0.0512 (6)	
C5A	1.1443 (2)	−0.01490 (19)	0.29002 (13)	0.0497 (5)	
H5A	1.1169	−0.0824	0.3094	0.060*	
C6A	1.0360 (2)	−0.01290 (17)	0.22340 (12)	0.0449 (5)	
H6A	1.0759	0.0428	0.1971	0.054*	
C7A	0.7816 (3)	0.06039 (18)	0.19605 (12)	0.0475 (5)	
C8A	0.8334 (3)	0.0573 (2)	0.12192 (13)	0.0605 (6)	
H8A	0.8585	−0.0199	0.1022	0.073*	
H8B	0.9238	0.1036	0.1247	0.073*	
C9A	0.8240 (2)	−0.05747 (19)	0.35050 (11)	0.0453 (5)	
C10A	0.7107 (3)	−0.1336 (2)	0.32057 (13)	0.0574 (6)	
H10A	0.6485	−0.1167	0.2829	0.069*	
C11A	0.6880 (3)	−0.2336 (2)	0.34529 (14)	0.0631 (7)	
H11A	0.6117	−0.2835	0.3241	0.076*	
C12A	0.7778 (3)	−0.2598 (2)	0.40118 (13)	0.0563 (6)	
C13A	0.8878 (3)	−0.1850 (2)	0.43326 (13)	0.0579 (6)	
H13A	0.9478	−0.2014	0.4717	0.070*	
C14A	0.9092 (3)	−0.0844 (2)	0.40798 (12)	0.0551 (6)	
H14A	0.9833	−0.0336	0.4305	0.066*	
C15A	0.8486 (4)	−0.3965 (3)	0.47465 (18)	0.0905 (10)	
H15A	0.9501	−0.3984	0.4606	0.136*	
H15B	0.8193	−0.4706	0.4823	0.136*	
H15C	0.8420	−0.3432	0.5178	0.136*	
C16A	1.3063 (3)	−0.0307 (3)	0.27004 (17)	0.0746 (8)	
H16A	1.3329	0.0267	0.2434	0.112*	
H16B	1.3165	−0.1042	0.2417	0.112*	
H16C	1.3716	−0.0246	0.3124	0.112*	
C17A	1.0383 (2)	−0.12792 (17)	0.17620 (11)	0.0420 (5)	
C18A	1.1144 (3)	−0.1434 (2)	0.11456 (13)	0.0537 (6)	
H18A	1.1528	−0.0806	0.0986	0.064*	
C19A	1.1343 (3)	−0.2505 (2)	0.07620 (13)	0.0564 (6)	
H19A	1.1851	−0.2591	0.0347	0.068*	
C20A	1.0794 (3)	−0.34451 (19)	0.09928 (12)	0.0498 (5)	
C21A	0.9971 (3)	−0.33057 (19)	0.15832 (13)	0.0544 (6)	
H21A	0.9544	−0.3932	0.1727	0.065*	
C22A	0.9780 (3)	−0.22299 (19)	0.19627 (13)	0.0506 (5)	
H22A	0.9230	−0.2146	0.2365	0.061*	
C23A	1.0734 (4)	−0.5463 (2)	0.08604 (16)	0.0838 (9)	
H23A	1.1205	−0.5439	0.1328	0.126*	
H23B	1.1075	−0.6115	0.0549	0.126*	
H23C	0.9663	−0.5511	0.0880	0.126*	
Cl1B	0.18993 (8)	0.15752 (6)	0.06531 (4)	0.0739 (2)	
O1B	0.15794 (18)	0.24812 (15)	0.21225 (9)	0.0618 (5)	
O2B	0.2506 (2)	0.81846 (16)	0.42158 (11)	0.0777 (6)	
O3B	0.7357 (2)	0.31515 (16)	0.38623 (10)	0.0720 (5)	
O4B	0.5886 (3)	0.70404 (15)	0.05185 (10)	0.0773 (6)	
N1B	0.38311 (19)	0.33705 (14)	0.24239 (10)	0.0452 (4)	
C2B	0.3520 (3)	0.35249 (19)	0.31822 (12)	0.0491 (5)	
H2B	0.2580	0.3113	0.3210	0.059*	
C3B	0.4754 (3)	0.2927 (2)	0.35614 (14)	0.0587 (6)	
H3C	0.4591	0.3042	0.4065	0.070*	
H3D	0.4680	0.2119	0.3383	0.070*	
C4B	0.6295 (3)	0.33301 (19)	0.34681 (13)	0.0531 (6)	
C5B	0.6454 (2)	0.40160 (19)	0.28828 (13)	0.0512 (6)	
H5B	0.6176	0.4794	0.3076	0.061*	
C6B	0.5369 (2)	0.36252 (18)	0.22178 (12)	0.0462 (5)	
H6B	0.5764	0.2925	0.1955	0.055*	
C7B	0.2822 (3)	0.27392 (17)	0.19570 (12)	0.0472 (5)	
C8B	0.3329 (3)	0.2340 (2)	0.12212 (13)	0.0582 (6)	
H8C	0.4199	0.1860	0.1251	0.070*	
H8D	0.3630	0.2991	0.1020	0.070*	
C9B	0.3264 (2)	0.47558 (19)	0.35026 (11)	0.0460 (5)	
C10B	0.2110 (3)	0.5346 (2)	0.32129 (13)	0.0567 (6)	
H10B	0.1481	0.4968	0.2841	0.068*	
C11B	0.1870 (3)	0.6480 (2)	0.34620 (14)	0.0630 (7)	
H11B	0.1093	0.6857	0.3255	0.076*	
C12B	0.2780 (3)	0.7051 (2)	0.40158 (13)	0.0571 (6)	
C13B	0.3896 (3)	0.6475 (2)	0.43301 (13)	0.0592 (6)	
H13B	0.4497	0.6847	0.4714	0.071*	
C14B	0.4119 (3)	0.5341 (2)	0.40725 (13)	0.0559 (6)	
H14B	0.4873	0.4960	0.4292	0.067*	
C15B	0.3483 (4)	0.8816 (3)	0.47489 (19)	0.0936 (10)	
H15D	0.3416	0.8530	0.5186	0.140*	
H15E	0.3202	0.9599	0.4817	0.140*	
H15F	0.4497	0.8747	0.4607	0.140*	
C16B	0.8068 (3)	0.4073 (3)	0.26829 (17)	0.0766 (8)	
H16D	0.8720	0.4253	0.3106	0.115*	
H16E	0.8165	0.4650	0.2395	0.115*	
H16F	0.8339	0.3354	0.2421	0.115*	
C17B	0.5366 (2)	0.45185 (17)	0.17413 (12)	0.0442 (5)	
C18B	0.6123 (3)	0.4349 (2)	0.11269 (13)	0.0562 (6)	
H18B	0.6545	0.3645	0.0979	0.067*	
C19B	0.6272 (3)	0.5191 (2)	0.07250 (14)	0.0610 (6)	
H19B	0.6778	0.5050	0.0310	0.073*	
C20B	0.5665 (3)	0.6245 (2)	0.09421 (13)	0.0531 (6)	
C21B	0.4870 (3)	0.6429 (2)	0.15428 (14)	0.0560 (6)	
H21B	0.4429	0.7128	0.1684	0.067*	
C22B	0.4730 (3)	0.55669 (19)	0.19369 (13)	0.0513 (6)	
H22B	0.4195	0.5699	0.2344	0.062*	
C23B	0.5585 (4)	0.8176 (2)	0.08017 (17)	0.0823 (9)	
H23D	0.4528	0.8258	0.0862	0.124*	
H23E	0.5889	0.8660	0.0483	0.124*	
H23F	0.6134	0.8383	0.1254	0.124*	

### Atomic displacement parameters (Å^2^)

**Table:**

	*U*^11^	*U*^22^	*U*^33^	*U*^12^	*U*^13^	*U*^23^
Cl1A	0.0836 (5)	0.0706 (4)	0.0615 (4)	0.0252 (3)	0.0072 (3)	0.0245 (3)
O1A	0.0493 (10)	0.0767 (12)	0.0659 (11)	0.0196 (8)	0.0120 (8)	0.0209 (9)
O2A	0.0729 (12)	0.0712 (12)	0.0928 (14)	−0.0160 (10)	−0.0030 (11)	0.0344 (11)
O3A	0.0686 (12)	0.0707 (12)	0.0683 (12)	−0.0169 (9)	−0.0010 (10)	0.0023 (9)
O4A	0.0953 (14)	0.0449 (9)	0.0646 (11)	0.0087 (9)	0.0128 (10)	−0.0055 (8)
N1A	0.0456 (10)	0.0415 (9)	0.0482 (11)	0.0085 (8)	0.0088 (8)	0.0096 (8)
C2A	0.0477 (13)	0.0493 (12)	0.0506 (13)	0.0117 (10)	0.0096 (10)	0.0045 (10)
C3A	0.0674 (16)	0.0426 (12)	0.0602 (15)	0.0061 (11)	0.0056 (12)	−0.0012 (11)
C4A	0.0565 (14)	0.0390 (11)	0.0591 (14)	−0.0064 (10)	0.0023 (11)	0.0121 (10)
C5A	0.0453 (12)	0.0427 (12)	0.0615 (14)	0.0036 (9)	0.0046 (11)	0.0092 (10)
C6A	0.0423 (12)	0.0388 (11)	0.0557 (13)	0.0040 (9)	0.0111 (10)	0.0112 (9)
C7A	0.0490 (13)	0.0394 (11)	0.0559 (14)	0.0040 (9)	0.0086 (11)	0.0114 (10)
C8A	0.0552 (14)	0.0681 (16)	0.0647 (16)	0.0121 (12)	0.0104 (12)	0.0272 (13)
C9A	0.0417 (12)	0.0519 (12)	0.0427 (12)	0.0065 (9)	0.0106 (9)	0.0063 (10)
C10A	0.0425 (13)	0.0775 (17)	0.0544 (14)	−0.0012 (11)	−0.0010 (11)	0.0193 (13)
C11A	0.0517 (14)	0.0775 (18)	0.0613 (16)	−0.0163 (13)	−0.0013 (12)	0.0173 (13)
C12A	0.0520 (14)	0.0608 (15)	0.0587 (15)	−0.0044 (11)	0.0087 (12)	0.0158 (12)
C13A	0.0590 (15)	0.0670 (16)	0.0490 (14)	−0.0031 (12)	−0.0023 (11)	0.0158 (12)
C14A	0.0587 (15)	0.0570 (14)	0.0477 (13)	−0.0064 (11)	−0.0011 (11)	0.0053 (11)
C15A	0.101 (2)	0.085 (2)	0.096 (2)	−0.0094 (18)	−0.0058 (19)	0.0486 (19)
C16A	0.0462 (14)	0.0823 (19)	0.090 (2)	0.0076 (13)	0.0011 (14)	0.0000 (16)
C17A	0.0397 (11)	0.0389 (11)	0.0479 (12)	0.0058 (8)	0.0077 (9)	0.0069 (9)
C18A	0.0614 (15)	0.0453 (12)	0.0576 (15)	0.0022 (10)	0.0159 (12)	0.0131 (11)
C19A	0.0677 (16)	0.0535 (14)	0.0495 (14)	0.0022 (11)	0.0180 (12)	0.0073 (11)
C20A	0.0561 (14)	0.0437 (12)	0.0476 (13)	0.0075 (10)	0.0035 (11)	0.0023 (10)
C21A	0.0629 (15)	0.0389 (12)	0.0624 (15)	−0.0046 (10)	0.0107 (12)	0.0086 (10)
C22A	0.0529 (13)	0.0452 (12)	0.0551 (14)	−0.0002 (10)	0.0176 (11)	0.0074 (10)
C23A	0.132 (3)	0.0422 (14)	0.0745 (19)	0.0136 (15)	−0.0016 (18)	0.0047 (13)
Cl1B	0.0847 (5)	0.0642 (4)	0.0679 (4)	−0.0272 (3)	0.0132 (4)	−0.0059 (3)
O1B	0.0498 (10)	0.0651 (11)	0.0693 (11)	−0.0137 (8)	0.0118 (8)	0.0053 (8)
O2B	0.0761 (13)	0.0598 (11)	0.0915 (14)	0.0171 (9)	−0.0023 (11)	−0.0006 (10)
O3B	0.0679 (12)	0.0776 (12)	0.0729 (12)	0.0189 (9)	−0.0030 (10)	0.0221 (10)
O4B	0.1166 (16)	0.0590 (11)	0.0629 (11)	−0.0122 (11)	0.0186 (11)	0.0247 (9)
N1B	0.0441 (10)	0.0406 (9)	0.0524 (11)	−0.0041 (7)	0.0094 (8)	0.0097 (8)
C2B	0.0477 (13)	0.0494 (12)	0.0535 (14)	−0.0056 (10)	0.0096 (10)	0.0164 (10)
C3B	0.0702 (16)	0.0482 (13)	0.0621 (15)	−0.0013 (11)	0.0054 (13)	0.0214 (11)
C4B	0.0587 (14)	0.0409 (12)	0.0597 (15)	0.0095 (10)	0.0025 (12)	0.0091 (10)
C5B	0.0459 (13)	0.0451 (12)	0.0640 (15)	0.0017 (10)	0.0046 (11)	0.0131 (11)
C6B	0.0421 (12)	0.0402 (11)	0.0577 (14)	−0.0005 (9)	0.0108 (10)	0.0098 (10)
C7B	0.0478 (13)	0.0349 (10)	0.0596 (14)	0.0001 (9)	0.0083 (11)	0.0082 (10)
C8B	0.0526 (14)	0.0541 (14)	0.0656 (16)	−0.0057 (11)	0.0119 (12)	0.0008 (12)
C9B	0.0411 (12)	0.0550 (13)	0.0445 (12)	−0.0024 (10)	0.0120 (9)	0.0124 (10)
C10B	0.0422 (13)	0.0705 (16)	0.0555 (14)	0.0039 (11)	0.0018 (11)	0.0057 (12)
C11B	0.0536 (14)	0.0705 (17)	0.0635 (16)	0.0189 (12)	0.0004 (12)	0.0082 (13)
C12B	0.0514 (14)	0.0604 (15)	0.0599 (15)	0.0098 (11)	0.0110 (12)	0.0083 (12)
C13B	0.0601 (15)	0.0618 (15)	0.0520 (14)	0.0022 (12)	−0.0016 (12)	0.0013 (12)
C14B	0.0571 (14)	0.0602 (15)	0.0517 (14)	0.0078 (11)	0.0001 (11)	0.0145 (11)
C15B	0.101 (2)	0.0663 (19)	0.101 (2)	0.0106 (17)	−0.009 (2)	−0.0164 (17)
C16B	0.0470 (15)	0.097 (2)	0.090 (2)	−0.0051 (14)	0.0015 (14)	0.0308 (17)
C17B	0.0413 (11)	0.0410 (11)	0.0517 (13)	−0.0035 (9)	0.0083 (10)	0.0096 (9)
C18B	0.0637 (15)	0.0447 (12)	0.0604 (15)	−0.0017 (11)	0.0162 (12)	0.0052 (11)
C19B	0.0740 (17)	0.0560 (15)	0.0542 (15)	−0.0080 (12)	0.0215 (12)	0.0062 (12)
C20B	0.0623 (15)	0.0493 (13)	0.0499 (13)	−0.0106 (11)	0.0033 (11)	0.0150 (11)
C21B	0.0631 (15)	0.0436 (12)	0.0641 (16)	0.0052 (11)	0.0088 (12)	0.0153 (11)
C22B	0.0534 (13)	0.0483 (13)	0.0555 (14)	0.0037 (10)	0.0158 (11)	0.0137 (11)
C23B	0.104 (2)	0.0595 (17)	0.091 (2)	−0.0104 (16)	0.0120 (18)	0.0324 (16)

### Geometric parameters (Å, °)

**Table:**

Cl1A—C8A	1.762 (2)	Cl1B—C8B	1.768 (2)
O1A—C7A	1.223 (3)	O1B—C7B	1.225 (3)
O2A—C12A	1.375 (3)	O2B—C12B	1.372 (3)
O2A—C15A	1.417 (4)	O2B—C15B	1.412 (4)
O3A—C4A	1.205 (3)	O3B—C4B	1.215 (3)
O4A—C20A	1.362 (3)	O4B—C20B	1.372 (3)
O4A—C23A	1.420 (3)	O4B—C23B	1.408 (4)
N1A—C7A	1.359 (3)	N1B—C7B	1.358 (3)
N1A—C2A	1.477 (3)	N1B—C2B	1.476 (3)
N1A—C6A	1.494 (3)	N1B—C6B	1.496 (3)
C2A—C3A	1.517 (3)	C2B—C9B	1.520 (3)
C2A—C9A	1.521 (3)	C2B—C3B	1.525 (3)
C2A—H2A	0.9800	C2B—H2B	0.9800
C3A—C4A	1.499 (3)	C3B—C4B	1.486 (3)
C3A—H3A	0.9700	C3B—H3C	0.9700
C3A—H3B	0.9700	C3B—H3D	0.9700
C4A—C5A	1.511 (3)	C4B—C5B	1.510 (3)
C5A—C16A	1.521 (3)	C5B—C16B	1.516 (3)
C5A—C6A	1.553 (3)	C5B—C6B	1.553 (3)
C5A—H5A	0.9800	C5B—H5B	0.9800
C6A—C17A	1.514 (3)	C6B—C17B	1.518 (3)
C6A—H6A	0.9800	C6B—H6B	0.9800
C7A—C8A	1.515 (3)	C7B—C8B	1.507 (3)
C8A—H8A	0.9700	C8B—H8C	0.9700
C8A—H8B	0.9700	C8B—H8D	0.9700
C9A—C14A	1.380 (3)	C9B—C14B	1.376 (3)
C9A—C10A	1.387 (3)	C9B—C10B	1.388 (3)
C10A—C11A	1.378 (4)	C10B—C11B	1.382 (4)
C10A—H10A	0.9300	C10B—H10B	0.9300
C11A—C12A	1.374 (3)	C11B—C12B	1.376 (4)
C11A—H11A	0.9300	C11B—H11B	0.9300
C12A—C13A	1.369 (3)	C12B—C13B	1.375 (3)
C13A—C14A	1.388 (3)	C13B—C14B	1.383 (3)
C13A—H13A	0.9300	C13B—H13B	0.9300
C14A—H14A	0.9300	C14B—H14B	0.9300
C15A—H15A	0.9600	C15B—H15D	0.9600
C15A—H15B	0.9600	C15B—H15E	0.9600
C15A—H15C	0.9600	C15B—H15F	0.9600
C16A—H16A	0.9600	C16B—H16D	0.9600
C16A—H16B	0.9600	C16B—H16E	0.9600
C16A—H16C	0.9600	C16B—H16F	0.9600
C17A—C22A	1.379 (3)	C17B—C18B	1.379 (3)
C17A—C18A	1.384 (3)	C17B—C22B	1.380 (3)
C18A—C19A	1.383 (3)	C18B—C19B	1.378 (3)
C18A—H18A	0.9300	C18B—H18B	0.9300
C19A—C20A	1.376 (3)	C19B—C20B	1.382 (4)
C19A—H19A	0.9300	C19B—H19B	0.9300
C20A—C21A	1.373 (3)	C20B—C21B	1.375 (3)
C21A—C22A	1.383 (3)	C21B—C22B	1.387 (3)
C21A—H21A	0.9300	C21B—H21B	0.9300
C22A—H22A	0.9300	C22B—H22B	0.9300
C23A—H23A	0.9600	C23B—H23D	0.9600
C23A—H23B	0.9600	C23B—H23E	0.9600
C23A—H23C	0.9600	C23B—H23F	0.9600
			
C12A—O2A—C15A	117.9 (2)	C12B—O2B—C15B	118.1 (2)
C20A—O4A—C23A	118.0 (2)	C20B—O4B—C23B	117.1 (2)
C7A—N1A—C2A	117.15 (18)	C7B—N1B—C2B	116.88 (17)
C7A—N1A—C6A	121.55 (18)	C7B—N1B—C6B	121.37 (18)
C2A—N1A—C6A	119.48 (17)	C2B—N1B—C6B	119.69 (17)
N1A—C2A—C3A	107.47 (19)	N1B—C2B—C9B	113.03 (17)
N1A—C2A—C9A	112.76 (17)	N1B—C2B—C3B	107.54 (19)
C3A—C2A—C9A	116.32 (19)	C9B—C2B—C3B	115.62 (19)
N1A—C2A—H2A	106.6	N1B—C2B—H2B	106.7
C3A—C2A—H2A	106.6	C9B—C2B—H2B	106.7
C9A—C2A—H2A	106.6	C3B—C2B—H2B	106.7
C4A—C3A—C2A	113.41 (19)	C4B—C3B—C2B	113.53 (19)
C4A—C3A—H3A	108.9	C4B—C3B—H3C	108.9
C2A—C3A—H3A	108.9	C2B—C3B—H3C	108.9
C4A—C3A—H3B	108.9	C4B—C3B—H3D	108.9
C2A—C3A—H3B	108.9	C2B—C3B—H3D	108.9
H3A—C3A—H3B	107.7	H3C—C3B—H3D	107.7
O3A—C4A—C3A	121.6 (2)	O3B—C4B—C3B	121.3 (2)
O3A—C4A—C5A	122.0 (2)	O3B—C4B—C5B	122.0 (2)
C3A—C4A—C5A	116.36 (19)	C3B—C4B—C5B	116.6 (2)
C4A—C5A—C16A	112.2 (2)	C4B—C5B—C16B	112.1 (2)
C4A—C5A—C6A	113.58 (18)	C4B—C5B—C6B	113.52 (18)
C16A—C5A—C6A	111.3 (2)	C16B—C5B—C6B	111.3 (2)
C4A—C5A—H5A	106.4	C4B—C5B—H5B	106.5
C16A—C5A—H5A	106.4	C16B—C5B—H5B	106.5
C6A—C5A—H5A	106.4	C6B—C5B—H5B	106.5
N1A—C6A—C17A	113.52 (17)	N1B—C6B—C17B	112.70 (17)
N1A—C6A—C5A	111.62 (18)	N1B—C6B—C5B	111.29 (18)
C17A—C6A—C5A	108.61 (17)	C17B—C6B—C5B	108.99 (17)
N1A—C6A—H6A	107.6	N1B—C6B—H6B	107.9
C17A—C6A—H6A	107.6	C17B—C6B—H6B	107.9
C5A—C6A—H6A	107.6	C5B—C6B—H6B	107.9
O1A—C7A—N1A	123.0 (2)	O1B—C7B—N1B	122.5 (2)
O1A—C7A—C8A	121.1 (2)	O1B—C7B—C8B	121.2 (2)
N1A—C7A—C8A	115.8 (2)	N1B—C7B—C8B	116.25 (19)
C7A—C8A—Cl1A	112.47 (17)	C7B—C8B—Cl1B	112.11 (16)
C7A—C8A—H8A	109.1	C7B—C8B—H8C	109.2
Cl1A—C8A—H8A	109.1	Cl1B—C8B—H8C	109.2
C7A—C8A—H8B	109.1	C7B—C8B—H8D	109.2
Cl1A—C8A—H8B	109.1	Cl1B—C8B—H8D	109.2
H8A—C8A—H8B	107.8	H8C—C8B—H8D	107.9
C14A—C9A—C10A	116.9 (2)	C14B—C9B—C10B	116.8 (2)
C14A—C9A—C2A	123.3 (2)	C14B—C9B—C2B	123.9 (2)
C10A—C9A—C2A	119.7 (2)	C10B—C9B—C2B	119.4 (2)
C11A—C10A—C9A	121.6 (2)	C11B—C10B—C9B	121.8 (2)
C11A—C10A—H10A	119.2	C11B—C10B—H10B	119.1
C9A—C10A—H10A	119.2	C9B—C10B—H10B	119.1
C12A—C11A—C10A	120.2 (2)	C12B—C11B—C10B	119.9 (2)
C12A—C11A—H11A	119.9	C12B—C11B—H11B	120.0
C10A—C11A—H11A	119.9	C10B—C11B—H11B	120.0
C13A—C12A—C11A	119.7 (2)	O2B—C12B—C13B	124.1 (2)
C13A—C12A—O2A	124.0 (2)	O2B—C12B—C11B	116.5 (2)
C11A—C12A—O2A	116.3 (2)	C13B—C12B—C11B	119.4 (2)
C12A—C13A—C14A	119.5 (2)	C12B—C13B—C14B	119.8 (2)
C12A—C13A—H13A	120.3	C12B—C13B—H13B	120.1
C14A—C13A—H13A	120.3	C14B—C13B—H13B	120.1
C9A—C14A—C13A	122.1 (2)	C9B—C14B—C13B	122.2 (2)
C9A—C14A—H14A	119.0	C9B—C14B—H14B	118.9
C13A—C14A—H14A	119.0	C13B—C14B—H14B	118.9
O2A—C15A—H15A	109.5	O2B—C15B—H15D	109.5
O2A—C15A—H15B	109.5	O2B—C15B—H15E	109.5
H15A—C15A—H15B	109.5	H15D—C15B—H15E	109.5
O2A—C15A—H15C	109.5	O2B—C15B—H15F	109.5
H15A—C15A—H15C	109.5	H15D—C15B—H15F	109.5
H15B—C15A—H15C	109.5	H15E—C15B—H15F	109.5
C5A—C16A—H16A	109.5	C5B—C16B—H16D	109.5
C5A—C16A—H16B	109.5	C5B—C16B—H16E	109.5
H16A—C16A—H16B	109.5	H16D—C16B—H16E	109.5
C5A—C16A—H16C	109.5	C5B—C16B—H16F	109.5
H16A—C16A—H16C	109.5	H16D—C16B—H16F	109.5
H16B—C16A—H16C	109.5	H16E—C16B—H16F	109.5
C22A—C17A—C18A	117.4 (2)	C18B—C17B—C22B	117.4 (2)
C22A—C17A—C6A	121.5 (2)	C18B—C17B—C6B	120.8 (2)
C18A—C17A—C6A	120.81 (19)	C22B—C17B—C6B	121.5 (2)
C19A—C18A—C17A	121.2 (2)	C19B—C18B—C17B	121.9 (2)
C19A—C18A—H18A	119.4	C19B—C18B—H18B	119.0
C17A—C18A—H18A	119.4	C17B—C18B—H18B	119.0
C20A—C19A—C18A	120.2 (2)	C18B—C19B—C20B	119.7 (2)
C20A—C19A—H19A	119.9	C18B—C19B—H19B	120.2
C18A—C19A—H19A	119.9	C20B—C19B—H19B	120.2
O4A—C20A—C21A	124.9 (2)	O4B—C20B—C21B	124.4 (2)
O4A—C20A—C19A	115.7 (2)	O4B—C20B—C19B	116.0 (2)
C21A—C20A—C19A	119.4 (2)	C21B—C20B—C19B	119.6 (2)
C20A—C21A—C22A	119.7 (2)	C20B—C21B—C22B	119.6 (2)
C20A—C21A—H21A	120.1	C20B—C21B—H21B	120.2
C22A—C21A—H21A	120.1	C22B—C21B—H21B	120.2
C17A—C22A—C21A	121.9 (2)	C17B—C22B—C21B	121.7 (2)
C17A—C22A—H22A	119.1	C17B—C22B—H22B	119.1
C21A—C22A—H22A	119.1	C21B—C22B—H22B	119.1
O4A—C23A—H23A	109.5	O4B—C23B—H23D	109.5
O4A—C23A—H23B	109.5	O4B—C23B—H23E	109.5
H23A—C23A—H23B	109.5	H23D—C23B—H23E	109.5
O4A—C23A—H23C	109.5	O4B—C23B—H23F	109.5
H23A—C23A—H23C	109.5	H23D—C23B—H23F	109.5
H23B—C23A—H23C	109.5	H23E—C23B—H23F	109.5
			
C7A—N1A—C2A—C3A	−114.3 (2)	C7B—N1B—C2B—C9B	−116.8 (2)
C6A—N1A—C2A—C3A	50.6 (2)	C6B—N1B—C2B—C9B	79.3 (2)
C7A—N1A—C2A—C9A	116.2 (2)	C7B—N1B—C2B—C3B	114.3 (2)
C6A—N1A—C2A—C9A	−78.9 (2)	C6B—N1B—C2B—C3B	−49.6 (2)
N1A—C2A—C3A—C4A	−57.1 (3)	N1B—C2B—C3B—C4B	56.5 (3)
C9A—C2A—C3A—C4A	70.4 (3)	C9B—C2B—C3B—C4B	−70.9 (3)
C2A—C3A—C4A—O3A	−162.9 (2)	C2B—C3B—C4B—O3B	162.0 (2)
C2A—C3A—C4A—C5A	15.6 (3)	C2B—C3B—C4B—C5B	−15.2 (3)
O3A—C4A—C5A—C16A	−19.6 (3)	O3B—C4B—C5B—C16B	20.4 (3)
C3A—C4A—C5A—C16A	161.9 (2)	C3B—C4B—C5B—C16B	−162.4 (2)
O3A—C4A—C5A—C6A	−146.9 (2)	O3B—C4B—C5B—C6B	147.6 (2)
C3A—C4A—C5A—C6A	34.7 (3)	C3B—C4B—C5B—C6B	−35.2 (3)
C7A—N1A—C6A—C17A	−74.5 (2)	C7B—N1B—C6B—C17B	75.1 (2)
C2A—N1A—C6A—C17A	121.3 (2)	C2B—N1B—C6B—C17B	−121.8 (2)
C7A—N1A—C6A—C5A	162.34 (18)	C7B—N1B—C6B—C5B	−162.16 (19)
C2A—N1A—C6A—C5A	−1.9 (2)	C2B—N1B—C6B—C5B	1.0 (3)
C4A—C5A—C6A—N1A	−41.6 (2)	C4B—C5B—C6B—N1B	42.3 (3)
C16A—C5A—C6A—N1A	−169.42 (19)	C16B—C5B—C6B—N1B	169.9 (2)
C4A—C5A—C6A—C17A	−167.52 (18)	C4B—C5B—C6B—C17B	167.23 (19)
C16A—C5A—C6A—C17A	64.7 (2)	C16B—C5B—C6B—C17B	−65.2 (2)
C2A—N1A—C7A—O1A	−13.2 (3)	C2B—N1B—C7B—O1B	13.3 (3)
C6A—N1A—C7A—O1A	−177.7 (2)	C6B—N1B—C7B—O1B	176.9 (2)
C2A—N1A—C7A—C8A	166.74 (19)	C2B—N1B—C7B—C8B	−165.06 (19)
C6A—N1A—C7A—C8A	2.2 (3)	C6B—N1B—C7B—C8B	−1.5 (3)
O1A—C7A—C8A—Cl1A	0.2 (3)	O1B—C7B—C8B—Cl1B	3.9 (3)
N1A—C7A—C8A—Cl1A	−179.68 (16)	N1B—C7B—C8B—Cl1B	−177.72 (16)
N1A—C2A—C9A—C14A	121.4 (2)	N1B—C2B—C9B—C14B	−121.1 (2)
C3A—C2A—C9A—C14A	−3.4 (3)	C3B—C2B—C9B—C14B	3.5 (3)
N1A—C2A—C9A—C10A	−57.8 (3)	N1B—C2B—C9B—C10B	58.8 (3)
C3A—C2A—C9A—C10A	177.4 (2)	C3B—C2B—C9B—C10B	−176.7 (2)
C14A—C9A—C10A—C11A	−2.5 (4)	C14B—C9B—C10B—C11B	2.8 (4)
C2A—C9A—C10A—C11A	176.8 (2)	C2B—C9B—C10B—C11B	−177.0 (2)
C9A—C10A—C11A—C12A	0.5 (4)	C9B—C10B—C11B—C12B	−0.6 (4)
C10A—C11A—C12A—C13A	1.4 (4)	C15B—O2B—C12B—C13B	3.9 (4)
C10A—C11A—C12A—O2A	−177.3 (2)	C15B—O2B—C12B—C11B	−175.7 (3)
C15A—O2A—C12A—C13A	−2.9 (4)	C10B—C11B—C12B—O2B	177.7 (2)
C15A—O2A—C12A—C11A	175.8 (3)	C10B—C11B—C12B—C13B	−1.9 (4)
C11A—C12A—C13A—C14A	−1.2 (4)	O2B—C12B—C13B—C14B	−177.6 (2)
O2A—C12A—C13A—C14A	177.4 (2)	C11B—C12B—C13B—C14B	2.0 (4)
C10A—C9A—C14A—C13A	2.6 (4)	C10B—C9B—C14B—C13B	−2.8 (4)
C2A—C9A—C14A—C13A	−176.6 (2)	C2B—C9B—C14B—C13B	177.1 (2)
C12A—C13A—C14A—C9A	−0.8 (4)	C12B—C13B—C14B—C9B	0.4 (4)
N1A—C6A—C17A—C22A	−55.9 (3)	N1B—C6B—C17B—C18B	−131.7 (2)
C5A—C6A—C17A—C22A	68.8 (3)	C5B—C6B—C17B—C18B	104.2 (2)
N1A—C6A—C17A—C18A	130.6 (2)	N1B—C6B—C17B—C22B	54.4 (3)
C5A—C6A—C17A—C18A	−104.6 (2)	C5B—C6B—C17B—C22B	−69.6 (3)
C22A—C17A—C18A—C19A	−2.5 (3)	C22B—C17B—C18B—C19B	1.1 (3)
C6A—C17A—C18A—C19A	171.2 (2)	C6B—C17B—C18B—C19B	−173.0 (2)
C17A—C18A—C19A—C20A	−0.6 (4)	C17B—C18B—C19B—C20B	0.8 (4)
C23A—O4A—C20A—C21A	−7.8 (4)	C23B—O4B—C20B—C21B	15.2 (4)
C23A—O4A—C20A—C19A	172.7 (2)	C23B—O4B—C20B—C19B	−166.2 (3)
C18A—C19A—C20A—O4A	−176.6 (2)	C18B—C19B—C20B—O4B	178.9 (2)
C18A—C19A—C20A—C21A	3.8 (4)	C18B—C19B—C20B—C21B	−2.4 (4)
O4A—C20A—C21A—C22A	176.6 (2)	O4B—C20B—C21B—C22B	−179.3 (2)
C19A—C20A—C21A—C22A	−3.8 (4)	C19B—C20B—C21B—C22B	2.2 (4)
C18A—C17A—C22A—C21A	2.5 (3)	C18B—C17B—C22B—C21B	−1.4 (3)
C6A—C17A—C22A—C21A	−171.2 (2)	C6B—C17B—C22B—C21B	172.7 (2)
C20A—C21A—C22A—C17A	0.7 (4)	C20B—C21B—C22B—C17B	−0.3 (4)

### Hydrogen-bond geometry (Å, °)

**Table:**

*D*—H···*A*	*D*—H	H···*A*	*D*···*A*	*D*—H···*A*
C3A—H3A···O3B	0.97	2.58	3.063 (3)	111
C6A—H6A···O1B^i^	0.98	2.53	3.350 (3)	142
C6B—H6B···O1A	0.98	2.54	3.363 (3)	142
C8A—H8B···Cl1B^i^	0.97	2.81	3.684 (3)	150

Symmetry codes:  (i) *x*+1, *y*, *z*.
